# Management of Amniotic Fluid Embolism (AFE) using anticoagulation-free ECMO

**DOI:** 10.1055/a-2685-9833

**Published:** 2025-09-05

**Authors:** Peijie Yan, Chuihua Sun, Xiaoyan Ma, Xin Sun, Liming Wang, Qinghai Zhang

**Affiliations:** 1 372527 Department of Critical Care Medicine, Weifang People's Hospital, Shandong Second Medical University, Weifang, China; 2 117907 School of Clinical Medicine, Shandong Second Medical University, Weifang, China

**Keywords:** Amniotic Fluid Embolism, disseminated intravascular coagulation, VA-ECMO, anticoagulation-free

## Abstract

Amniotic fluid embolism (AFE) is a critical obstetric complication characterized by the entry of amniotic fluid and its components into maternal circulation during parturition, leading to acute cardiopulmonary failure, disseminated intravascular coagulation (DIC), and anaphylactic shock. Affected patients typically exhibit abrupt onset, rapid progression, and exceedingly high mortality. Early recognition and prompt intervention are pivotal in AFE management. We present a case of AFE-induced cardiac arrest in a 35-year-old primigravida who developed acute cardiopulmonary collapse during vaginal delivery, followed by cardiac arrest. After cardiopulmonary resuscitation, massive transfusion, and crash emergency cesarean section, anticoagulant-free venoarterial extracorporeal membrane oxygenation (VA-ECMO) was initiated. Subsequent multimodal therapies – including aggressive transfusion support, uterine artery embolization for hemostasis, exploratory laparotomy, and targeted DIC management – ensured safe ECMO maintenance without device-related complications. By hospital day 3, hemodynamic and respiratory stability were achieved, enabling successful ECMO weaning. This case highlights that ECMO remains a viable therapeutic option for salvaging critically ill AFE patients with refractory hemorrhagic shock, DIC, and cardiopulmonary failure.

## Introduction

Amniotic fluid embolism (AFE) is a rare and fatal obstetric complication with a mortality rate as high as 43%. The pathophysiology of AFE involves the entry of amniotic fluid containing fetal cells and tissue into the maternal circulation, triggering severe respiratory and circulatory failure as well as hemorrhagic tendencies due to disseminated intravascular coagulation (DIC) with hyperfibrinolysis.

Venoarterial extracorporeal membrane oxygenation (VA-ECMO) has been considered for life-threatening cases; however, hemorrhage remains one of its complications. No current guidelines recommend extracorporeal membrane oxygenation (ECMO) for hemorrhagic shock secondary to AFE. Furthermore, the inability to administer anticoagulation is a contraindication for ECMO, and the use of anticoagulation-free ECMO in AFE patients remains controversial.

In this case report, we describe a fatal AFE case with respiratory/circulatory failure and DIC, where anticoagulation-free VA-ECMO was successfully utilized to manage the patient without severe complications.

## Case presentation

**Fig. 1:**
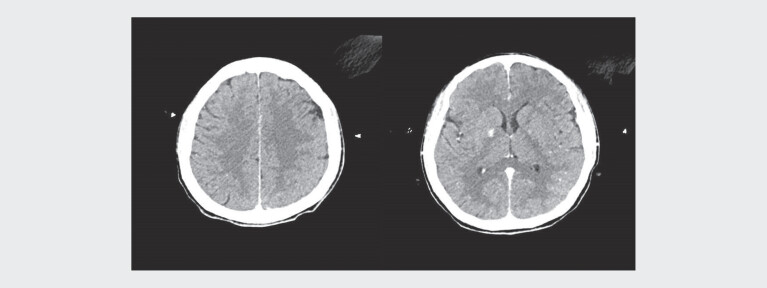
Cranial computed tomography manifestations 15 hours post-admission.

**Fig. 2:**
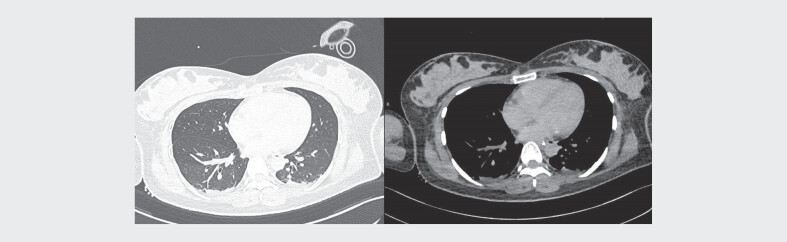
Thoracic computed tomography manifestations 15 hours post-admission.

A 35-year-old multipara with an uncomplicated pregnancy developed uterine contractions at 39 weeks of gestation. During vaginal delivery, she experienced sudden convulsions, cyanosis, persistent hypoxemia, and progressive fetal bradycardia, followed by ventricular fibrillation and cardiac arrest, suggestive of AFE. The local hospital immediately performed endotracheal intubation with mechanical ventilation, continuous cardiopulmonary resuscitation (CPR), and electrical defibrillation, while administering intravenous epinephrine, dexamethasone, and continuous infusions of norepinephrine and dopamine. The patient regained sinus rhythm after 20 minutes, after which a cesarean section was promptly performed, successfully delivering a healthy male infant. During resuscitation, persistent hemorrhage necessitated transfusion of 10 units of blood, 1,000 mL fresh frozen plasma, and 1 therapeutic unit of platelets. Post-resuscitation, the patient exhibited persistent cardiopulmonary dysfunction with severe myocardial injury but no significant neurological impairment, prompting initiation of VA-ECMO without systemic anticoagulation due to coagulopathy. The patient was subsequently transferred to our hospital for further multidisciplinary care. Cranial and thoracic CT imaging findings at 15 hours post-admission are presented in Fig. 1 and 2 . Serial laboratory parameters—including arterial blood gas analysis, serum biochemistry, blood cell counts and coagulation parameters – from admission to 60 hours post-admission are detailed in Table 1 2 3 4 .

**Table:** **Table 1** Changes in Arterial Blood Gas Analysis from Admission to 60 Hours Post-Admission.

	Upon ICU admission	at 4 h	at 10 h	at 24 h	at 32 h	at 36 h	at 48 h	at 60 h
pH	7.54	7.43	7.48	7.5	7.55	7.49	7.39	7.46
pCO2 (mmHg)	11	32	27	33	28	33	45	36
pO2 (mmHg)	542	189	188	164	61	144	98	123
Na+(mmol/L)	140	141	143	142	142	138	141	141
K+(mmol/L)	3.1	3.6	3	3.4	3.1	3.6	3.8	3.5
Ca++ (mmol/L)	0.87	1.14	1.2	1.24	1.08	1.18	1.23	1.15
Glu (mmol/L)	14.2	8.9	16.9	7.7	9.1	6.5	5.8	6.2
Lac (mmol/L)	11.1	6.3	5.4	4.3	3.6	2.9	0.9	0.8
HCO3- (mmol/L)	9.4	21.2	20.1	25.7	24.5	25.1	27.2	25.6
BE (mmol/L)	−11.6	−2.6	−2.6	2.4	2.2	1.8	1.9	0.8
SO2 (%)	100	100	100	100	94	99	98	99
Hb (g/dL)	7.1	9.2	9.2	7.1	7.5	7.8	8.5	6.9

**Table:** **Table 2** Changes in Serum Biochemistry Parameters from Admission to 60 Hours Post-Admission.

	Upon ICU admission	at 10 h	at 32 h	at 36 h	at 60 h
ALT (U/L)	27	26	20	21	30
AST (U/L)	64	65	47	46	57
TBIL (ummol/L)	34.7	40.1	28.4	28.3	27
DBIL (ummol/L)	19.5	21.3	11.1	11.3	10.6
IBIL (ummol/L)	15.3	18.8	17.3	17	16.4
TP (g/L)	34.5	46.5	47.4	52.5	58.6
ALB (g/L)	21.6	30.9	31.7	32.7	38.8
BUN (mmol/L)	4.3	6.8	3.6	4.2	4.7
CR (ummol/L)	62	74	54	49	56
CK (U/L)	551		634	764	900
CK-MB (ng/mL)	39.56		12.07	7.53	3.37
LDH (U/L)	403		353	381	393
AMY (U/L)	461		440	472	110

**Table:** **Table 3** Changes in Blood Cell Counts from Admission to 60 Hours Post-Admission *(White Blood Cell Count, Red Blood Cell Count, and Platelet Count only).*

	Upon ICU admission	at 4 h	at 10 h	at 32 h	at 48 h	at 60 h
RBC (×10^12/L)	2.34	3.29	2.51	2.55	3.33	3.39
WBC (×10^9/L)	15.84	12.72	19.62	12.5	13.45	14.17
PLT (×10^9/L)	76	82	82	94	67	79

**Table:** **Table 4** Changes in Coagulation Parameters from Admission to 60 Hours Post-Admission.

	Upon ICU admission	at 4 h	at 10 h	at 32 h	at 36 h	at 48 h	at 60 h
PT (S)	13.3	12.9	12.9	11.9	11.2	11.1	10.5
FIB (g/L)	0.86	1.28	2.47	2.39	2.68	3.52	4.17
TT (S)	43.9	29.2	19.9	16.3	17.5	16.6	16.1
APTT (S)	47.1	44.7	53.1	44.6	38.2	37.5	34.6
D-Dimer (ug/ml)	>128		57.05	20.12	14.61		13.99

Upon ICU admission, the patient required high-dose norepinephrine (2 μg/kg/h) to maintain blood pressure, with arterial blood gas analysis indicating severe acidosis and microcirculatory dysfunction (lactate 11.1 mmol/L, HCO₃- 3.4 mmol/L, BE –11.6 mmol/L). Concurrently, ionized calcium (Ca²-) levels dropped to 0.87 mmol/L, reflecting calcium depletion as a coagulation factor during DIC. Coagulation studies confirmed ongoing DIC, with D-dimer>128 μg/mL, fibrinogen 0.86 g/L, and markedly prolonged TT and APTT. Despite pre-ICU transfusions (10 units of blood, 1 therapeutic unit of platelets, and 1,000 mL plasma), persistent cytopenia was observed. High-sensitivity troponin I (>4802.4 pg/mL) indicated severe myocardial injury. Systemic oxygen saturation was stabilized under VA-ECMO support.

At 10 hours post-ICU admission, bedside ultrasound revealed increasing peritoneal fluid. Despite transfusion of 7.5 units of blood post-admission, hemoglobin levels remained suboptimally elevated (9.2 g/dL), prompting suspicion of active hemorrhage. The patient underwent emergency pelvic angiography with uterine artery embolization (UAE); however, no active contrast extravasation was detected. Intraoperative transfusions included 6 units of blood, 1 therapeutic unit of platelets, 1,350 mL fresh frozen plasma, and 18.5 units of cryoprecipitate. Despite this, hemoglobin continued to decline (Hb 7.1 g/dL). Exploratory laparotomy at 32 hours post-admission identified bleeding from a ruptured pelvic vessel, which was surgically ligated. Following this intervention, no further bleeding occurred, and hemodynamic parameters stabilized.

By 36 hours post-ICU admission, the patient exhibited marked improvement in hemorrhagic shock, coagulation profile (fibrinogen 2.68 g/L, Hb 7.8 g/dL), and cardiopulmonary function, allowing VA-ECMO decannulation. Hemoglobin remained stable for 72 hours post-ECMO removal. Tracheal extubation was performed on day 5 of ICU admission following resolution of respiratory failure. A secondary infection (blood cultures: Escherichia coli) was treated with meropenem guided by antimicrobial susceptibility testing.

The patient was transferred to the obstetric ward on day 6 and successfully discharged on the eleventh day of admission.

## Discussion

AFE lacks specific laboratory diagnostic markers and is primarily diagnosed based on clinical manifestations after exclusion of alternative diagnoses. The classic clinical presentation involves the triad of hypoxemia, hypotension, and coagulopathy, with reported mortality rates ranging from 11% to 48% 1 . Survival rates decline significantly when cardiac arrest occurs 2 3 . As highlighted in a review by Pacheco et al., AFE remains a diagnosis of exclusion, and treatment principles focus on supportive care, including but not limited to advanced cardiovascular life support (ACLS) protocols, mechanical ventilation, massive transfusion protocols, hemodynamic stabilization with inotropes and vasopressors (e. g., norepinephrine), and immediate delivery of the fetus 4 . The critical determinant of outcomes in clinically suspected AFE is rapid recognition and multidisciplinary response, emphasizing timely intervention to address concurrent cardiorespiratory failure and coagulopathy.

The pathogenesis of AFE can be divided into three interrelated phases 5 . Phase 1 occurs within minutes of AFE onset: amniotic fluid components enter the maternal circulation via uterine trauma, mechanically obstructing pulmonary microvasculature, leading to acute pulmonary hypertension, right ventricular overload, and acute right heart failure. Concurrently, amniotic debris triggers massive release of inflammatory mediators (e. g., histamine, leukotrienes), causing systemic vasodilation, capillary leakage, and exacerbation of hypoxemia and shock. Pulmonary vasospasm is further amplified by prostaglandin F2α and serotonin​ released from damaged lung tissue. Clinically, this phase manifests as sudden dyspnea, coughing, cyanosis, hypotension, seizures, or loss of consciousness, with some patients progressing to sudden death within minutes 6 7 . Phase 2 involves activation of the extrinsic coagulation pathway by amniotic fluid-derived tissue factor (Factor III) and Factor X activators, resulting in widespread microthrombi formation, consumption of coagulation factors and platelets, and subsequent coagulopathy. Secondary hyperfibrinolysis generates elevated fibrin degradation products (FDPs), further destabilizing hemostasis and causing intractable postpartum hemorrhage 5 8 . Phase 3 ensues if early phases are inadequately managed: persistent hypotension, DIC-induced hypoperfusion, and systemic inflammatory response syndrome (SIRS) culminate in multiorgan failure, which is often fatal.

VA-ECMO is primarily indicated for severe cardiogenic shock or circulatory failure. While there are no absolute contraindications to VA-ECMO, its use in patients with active bleeding and uncorrected coagulopathy requires cautious evaluation. In AFE patients, early initiation of ECMO is critical to support oxygenation and circulation in cases of cardiopulmonary failure or cardiac arrest 2 9 . Studies suggest that 70% of AFE patients supported by VA-ECMO survive to discharge; however, these outcomes typically require partial correction of DIC, absence of active bleeding, and near-normal coagulation profiles prior to ECMO initiation 10 .

Before initiating ECMO in this patient, we comprehensively evaluated her clinical status. Following CPR and crash emergency cesarean section, her cardiopulmonary function remained critically impaired despite mechanical ventilation and high-dose vasopressors (norepinephrine: 2 μg/kg/min). The entry of amniotic fluid and fetal debris into the circulation triggered SIRS via pro-inflammatory mediators (e. g., IL-6, TNF-α), exacerbating shock and activating the coagulation cascade 11 12 . This resulted in widespread microthrombosis, consumption of coagulation factors and platelets, and hyperfibrinolysis, which further suppressed platelet aggregation. The combination of thrombocytopenia and hyperfibrinolysis significantly increased bleeding risk in this DIC patient 6 .

At the time of ECMO initiation, the patient was in the consumptive hypocoagulable phase of DIC, characterized by refractory hemorrhage. This phase represents a critical window for intervention in AFE. During ECMO therapy, blood contact with the ECMO circuit directly activates coagulation factor XII (FXII), triggering the intrinsic coagulation pathway. Concurrently, ECMO materials induce inflammatory responses that promote tissue factor (TF) release, activating the extrinsic coagulation pathway​ and leading to circuit thrombosis. Consequently, ECMO management guidelines indicate that systemic anticoagulation is typically required to prevent circuit thrombosis, with heparin and direct thrombin inhibitors (DTIs) being the most common anticoagulants for ECMO patients 13 . Current research emphasizes that ECMO anticoagulation monitoring requires comprehensive and standardized assessment using multiple coagulation parameters, including activated partial thromboplastin time (aPTT), anti-factor Xa (anti-Xa) levels, thromboelastography (TEG), antithrombin (AT) activity, platelet count, and fibrinogen concentration. These parameters must be adjusted according to the patient’s clinical hemostatic status and individualized risks for bleeding or thrombotic complications 14 .

Regardless of the type of anticoagulant or method used, their mechanism involves inhibiting both the intrinsic and extrinsic coagulation pathways, thereby reducing fibrin (thrombus) formation. In patients with disseminated intravascular coagulation (DIC) during the consumptive hypocoagulable phase, the prolonged coagulation time resulting from consumption of clotting factors represents a pathological state. Paradoxically, this achieves an effect analogous to therapeutic anticoagulation by suppressing blood coagulation. This phenomenon provides the pathophysiological rationale for implementing anticoagulation-free VA-ECMO in such cases 15 . And, in DIC patients, reduced antithrombin levels impair anticoagulant efficacy, while anticoagulation may exacerbate active bleeding. Conversely, reduced anticoagulation increases thrombotic risks. In AFE patients, coagulation factor deficiencies predispose to localized hematomas or hemorrhage, potentially escalating to fatal bleeding. A recent systematic review, however, found no increased risk of hemorrhagic or thrombotic complications in AFE patients managed with anticoagulation-free ECMO 9 .

In this case, we ultimately selected the widely utilized activated partial thromboplastin time (aPTT) combined with other coagulation parameters (fibrinogen, D-dimer, etc.) to monitor the patient&apos;s coagulation status. Based on fibrinogen levels and clotting factor assays, we made the paradoxical decision to administer coagulation factor replacement therapy to improve coagulopathy while implementing anticoagulation-free VA-ECMO. This approach was adopted despite uncorrected coagulopathy and ongoing active hemorrhage. Published studies support the safety of heparin-minimized or heparin-free strategies in ECMO patients with hemorrhagic risks. For instance, Moon et al. reported two cases of hemorrhagic shock with refractory acute respiratory distress syndrome (ARDS) requiring veno-venous extracorporeal membrane oxygenation (V-V ECMO). Both patients underwent minimal or no therapeutic anticoagulation during ECMO, tolerated multiple surgical interventions, and experienced no significant bleeding complications 16 . These findings suggest that ECMO can be cautiously considered in select patients with severe ARDS and high bleeding risks, provided meticulous hemostatic monitoring and transfusion support are maintained.

In patients with AFE-induced cardiac arrest, prompt cesarean section should be performed. During this case management, despite rapid preparation for surgery following cardiac arrest, a 20-minute interval elapsed from cardiac arrest onset to readiness for cesarean section. This represents a critical area for improvement identified through our experience 2 .

## Conclusion

AFE patients often present with hemorrhagic shock and DIC. VA-ECMO represents a viable therapeutic intervention for AFE-induced life-threatening circulatory and respiratory failure. In patients with coagulation-anticoagulation imbalance, aggressive transfusion protocols combined with anticoagulation-free ECMO may buy critical time for subsequent interventions. However, early application of anticoagulation-free ECMO requires meticulous assessment of coagulation profiles and hemorrhagic risks. The use of anticoagulation-free VA-ECMO in AFE management warrants further data validation through multicenter studies to confirm its efficacy and safety in this high-risk population.

## Notice

This article was changed according to the following Erratum on 25.09.2025.

## Erratum

In the above-mentioned article the following Funding Information has been added: This work was supported by Key Specialty Funds for the Intensive Care Medicine Department at Weifang People's Hospital for article processing charge. In addition, the first Affiliation has been corrected, the correct affiliation is: Department of Critical Care Medicine, Weifang People's Hospital, Shandong Second Medical University, Weifang, China. This was corrected in the online version on 25.9.2025
